# Extending the La Solubility Limit in Sr_3_Ir_2_O_7_ through High-Pressure High-Temperature Synthesis

**DOI:** 10.1021/acsmaterialsau.6c00140

**Published:** 2026-09-11

**Authors:** Cheng Peng, Weiwei Xie

**Affiliations:** Department of Chemistry, 3078 Michigan State University, East Lansing, Michigan 48864, United States

**Keywords:** high-pressure synthesis, Sr_3_Ir_2_O_7_, Ruddlesden−Popper oxides, lanthanum substitution, spin−lattice coupling, charge transport

## Abstract

La-doped bilayer iridates provide an important platform for studying the evolution of the spin-orbit-assisted Mott state under electron doping, but La substitution in conventional ambient-pressure-grown bulk samples has been reported only up to *x* ≈ 0.08. Here, we report the synthesis and physical properties of nominally La-doped (Sr_1–*x*_La_*x*_)_3_Ir_2_O_7_ (*x* = 0.05, 0.10, 0.15, and 0.20) prepared using high-pressure high-temperature techniques. Single-crystal X-ray diffraction refinements of selected crystals reveal significantly enhanced La incorporation, with nominal *x* = 0.05 and 0.15 corresponding to compositions of approximately (Sr_0.9_La_0.1_)_3_Ir_2_O_7_ and (Sr_0.8_La_0.2_)_3_Ir_2_O_7_, respectively. Scanning electron microscopy energy dispersive X-ray spectroscopy (SEM-EDS) confirms La incorporation and an overall increase in local La content across the series. At nominal *x* = 0.20, the bilayer phase instead transforms into cubic perovskite Sr_1–*x*_La_*x*_IrO_3_. The nominal *x* = 0.05 product exhibits a ferromagnetic-like transition near 186 K accompanied by magnetic hysteresis and subtle lattice anomalies indicative of spin–lattice coupling. Despite its high electron-doping level, the compound remains strongly insulating, consistent with a heavily doped localized magnetic insulating state distinct from both parent Sr_3_Ir_2_O_7_ and ambient-pressure La-doped samples. In contrast, the nominal *x* = 0.15 product displays metal-like electronic behavior, weakened magnetic order, and enhanced carrier delocalization, although disorder-driven localization persists at low temperatures. These results demonstrate that high-pressure synthesis extends the experimentally reported La-doping range of bilayer iridates and reveals electronic and magnetic states not previously reported through conventional synthesis routes.

## Graphical abstract

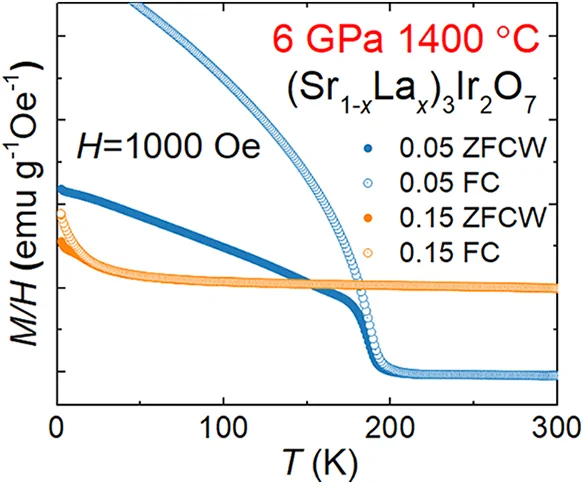

## Introduction

The layered iridates have emerged as an important platform for exploring the interplay of spin-orbit coupling, electron correlations, and lattice degrees of freedom.^1–6^ Among them, the bilayer Ruddlesden-Popper iridate Sr_3_Ir_2_O_7_ occupies a unique position near the boundary between a spin-orbit-assisted Mott insulator and a correlated metal.^7–10^ The delicate balance among Coulomb interactions, crystal-field effects, and strong spin-orbit coupling gives rise to a narrow-gap insulating state derived from the *J*_eff_ = 1/2 electronic configuration.^3,6^ Because Sr_3_Ir_2_O_7_ lies close to an electronic instability, small perturbations such as chemical substitution, pressure, or strain can strongly modify its magnetic and electronic ground states.^11,12^ Electron doping through La substitution on the Sr site has proven to be one of the most effective approaches for tuning the physical properties of Sr_3_Ir_2_O_7_.^11,13–16^ Previous studies on flux-grown single crystals of (Sr_1–*x*_La_*x*_)_3_Ir_2_O_7_ have shown that electron doping suppresses the parent antiferromagnetic insulating state and drives the system toward metallic behavior.^17^ However, previous studies of ambient-pressure-grown crystals have reported La substitution up to *x* ≈ 0.08.^18^ Within this narrow doping range, a rich set of phenomena has been observed, including the collapse of long-range antiferromagnetic order, electronic phase separation, persistent magnetic excitations, and unconventional metallic behavior.^11,13–18^ These results demonstrate that electron doping does not simply destroy the spin-orbit Mott state but instead produces competing electronic and magnetic phases governed by strong spin-charge-lattice coupling.

High-pressure synthesis provides an alternative strategy for accessing regions of the phase diagram beyond those previously reported under ambient-pressure conditions. High pressure has proven particularly effective for stabilizing metastable oxide phases, increasing dopant solubility, and modifying local bonding environments.^19–25^ In iridates, pressure can significantly alter Ir–O bond distances, Ir–O–Ir bond angles, and electronic bandwidths, thereby influencing both magnetic exchange interactions and charge transport.^26–28^ High-pressure high-temperature synthesis therefore provides an alternative route for stabilizing highly substituted oxide compositions..

In this work, we employ high-pressure high-temperature synthesis to investigate La-doped Sr_3_Ir_2_O_7_ beyond the doping range previously reported through conventional ambient-pressure methods. By combining single-crystal X-ray diffraction, SEM-EDS analysis, magnetization measurements, electrical transport, and variable-temperature structural characterization, we examine how enhanced La incorporation influences the structural, magnetic, and electronic properties of the bilayer iridate. Our study demonstrates that high-pressure synthesis yields La-rich Sr_3_Ir_2_O_7_ crystals beyond the previously reported doping range and allow us to investigate their magnetic and electronic properties.

## Experimental Methods

### High-Pressure Synthesis

Polycrystalline Sr_2_IrO_4_ was first prepared under ambient pressure by thoroughly mixing stoichiometric amounts of SrCO_3_ and IrO_2_, followed by pelletization and sequential heat treatments at 900, 1000, and 1100 °C with intermediate grinding steps. The resulting Sr_2_IrO_4_ powder was then mixed with La_2_O_3_ to achieve nominal compositions of (Sr_1–*x*_La_*x*_)_3_Ir_2_O_7_ (*x* = 0.05, 0.10, 0.15, and 0.20). The powder mixtures were loaded into Pt capsules and synthesized under high-pressure high-temperature conditions using a Walker-type multi-anvil press at the Lamont-Doherty Earth Observatory, Columbia University. For each synthesis, the Pt capsule was placed inside an Al_2_O_3_ crucible and surrounded by a LaCrO_3_ heater embedded within a Ceramacast 646 octahedral pressure medium. The assemblies were compressed to 6 GPa, heated to 1400 °C, and held at temperature for 24 h. After synthesis, the samples were cooled to room temperature before releasing pressure. The resulting products are denoted TT-1441 (nominal *x* = 0.05), BB-1475 (nominal *x* = 0.10), TT-1442 (nominal *x* = 0.15), and TT-1445 (nominal *x* = 0.20).

### Phase Analysis and Chemical Composition

Crystalline phase identity and lattice parameters were examined by powder X-ray diffraction (PXRD) using a Rigaku MiniFlex 600 diffractometer equipped with Cu Kα radiation (λ = 1.5406 Å). Diffraction data were collected at room temperature over the 2θ range of 5-90° with a step size of 0.01° and a scan rate of 1° min^–1^. Lattice parameters and profile agreement indices were obtained through Le Bail refinements using the GSAS-II software suite.^29^ Chemical compositions were evaluated by scanning electron microscopy (SEM) and energy-dispersive X-ray spectroscopy (EDS) using a JEOL JSM-6610LV electron microscope. Representative Sr–La–Ir–O regions were selected from backscattered-electron images while avoiding visibly distinct impurity regions. The EDS results are treated as semi-quantitative local composition estimates.

### Single-Crystal Structure Determination

Single-crystal X-ray diffraction measurements were performed on a Rigaku Synergy-S diffractometer equipped with Mo Kα radiation (λ = 0.71073 Å). Several crystals from each synthesis product were screened for diffraction quality. Full single-crystal X-ray diffraction datasets were collected from two independently selected crystals from the nominal *x* = 0.05 product, one crystal from the nominal *x* = 0.15 product, and one crystal from the nominal *x* = 0.20 product. For the nominal *x* = 0.05 product, one crystal was measured at 293 K and a second independent crystal was measured at 100, 150, 200, and 300 K. Data collection and reduction were carried out using the CrysAlisPro software package. Analytical absorption corrections based on indexed crystal faces and measured crystal dimensions were applied because of the high linear absorption coefficients. Crystal structures were solved by direct methods and refined by full-matrix least-squares techniques on F^2^ using the SHELXTL program suite.^30,31^ Variable-temperature single-crystal diffraction measurements were performed between 100 and 300 K to investigate structural evolution across the magnetic transition.

### Physical Property Measurements

Temperature- and field-dependent magnetic measurements were performed using a Quantum Design Magnetic Property Measurement System (MPMS3) in the temperature range of 2–300 K under magnetic fields up to 7 T. Electrical transport measurements were carried out using a Quantum Design DynaCool employing a standard four-probe configuration. Platinum wires were attached using silver paste for resistance measurements. Temperature-dependent resistance was measured between 2 and 300 K under applied magnetic fields up to 9 T.

## Results and Discussion

### Phase Identification and Chemical Compositions

The crystalline phase identity and structural evolution of the high-pressure synthesized La-doped Sr_3_Ir_2_O_7_ series were examined by powder X-ray diffraction (PXRD), and the results are shown in Figure 1 and Table S1. For the nominal *x* = 0.05 composition, all diffraction peaks can be indexed using the tetragonal *I*4/*mmm* structure of the bilayer Ruddlesden-Popper phase. The diffraction pattern is consistent with the single-crystal refinement discussed below and no impurity peaks are detected within the resolution of the measurement, indicating the crystallographic dominance of the La-doped bilayer iridate phase. For the nominal *x* = 0.10 composition, the diffraction pattern remains dominated by the bilayer *I*4/*mmm* phase, with peak positions closely matching those of the La-substituted Sr_3_Ir_2_O_7_ structure. However, several additional reflections become visible, most notably near 2θ ≈ 28° and 44°, indicating the presence of a secondary phase, although the bilayer phase remains the major component. Interestingly, for the nominal *x* = 0.15 composition, the diffraction pattern continues to be dominated by the bilayer phase, while the impurity reflections become noticeably weaker than those observed for *x* = 0.10. This reduction suggests improved stabilization of the bilayer structure under these synthesis conditions despite the higher La loading. Combined with the single-crystal refinements discussed below, these results demonstrate that high-pressure synthesis produced La-rich bilayer crystals with local La contents beyond those previously reported for ambient-pressure-grown crystals. A dramatic structural transformation occurs at the nominal *x* = 0.20 composition. The diffraction pattern of the major phase can no longer be indexed using the bilayer *I*4/*mmm* structure and instead is consistent with a cubic perovskite phase. Single-crystal diffraction identifies this phase to be Sr_0.4_La_0.6_IrO_3_ with space group *Pm*3̅*m*, as shown in Tables S2 and S3. The disappearance of the characteristic bilayer reflections and emergence of the cubic perovskite pattern indicate that the Sr_3_Ir_2_O_7_ framework is no longer the thermodynamic ground state at this high La concentration.

**Figure 1:**
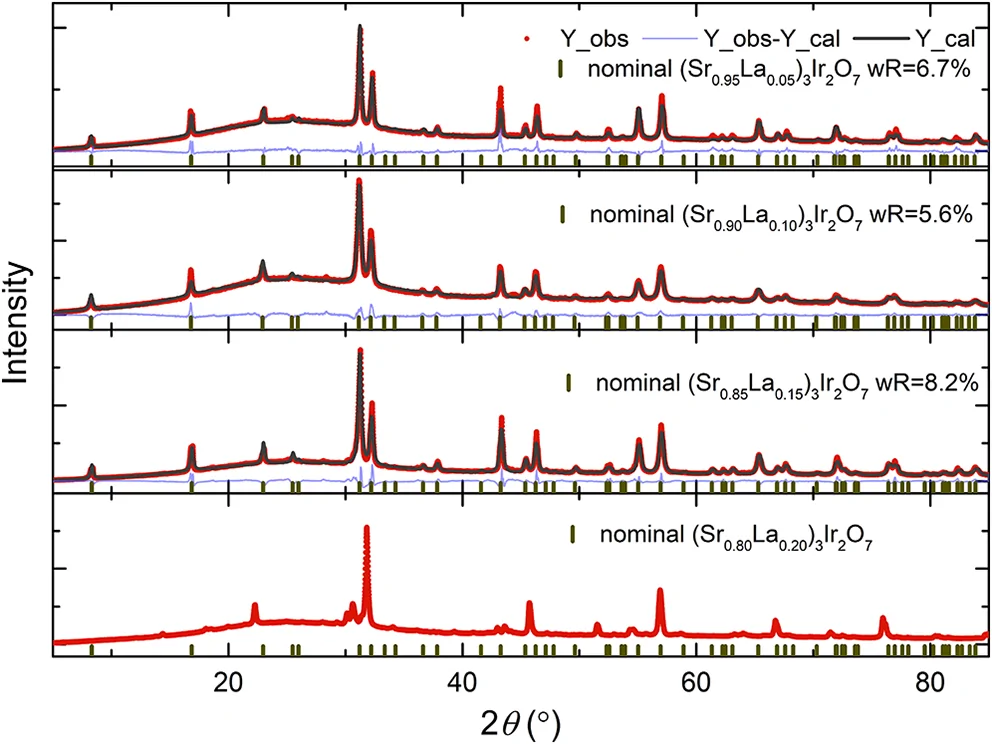
Powder X-ray diffraction patterns and Le Bail refinements of high-pressure synthesized La-doped Sr_3_Ir_2_O_7_ with nominal compositions *x* = 0.05, 0.10, 0.15, and 0.20. (Red circles represent experimental data, black lines represent the refinement, blue lines show the difference curve, and green tick marks indicate Bragg reflection positions.).

Single-crystal X-ray diffraction refinements on *x* = 0.05 and 0.15 compositions at 293 K presented in Tables 1 and 2 reveal that both compounds adopt the tetragonal *I*4/*mmm* space group characteristic of Sr_3_Ir_2_O_7_ shown in Figure 2a. The refinements converge with excellent reliability factors (*R*_1_ < 0.03), confirming the high crystallinity of the selected crystals and the suitability of the structural model. A notable result of the structural refinements is the high local La content observed in the selected crystals.. The nominal *x* = 0.05 and *x* = 0.15 samples refine to compositions of (Sr_0.9_La_0.1_)_3_Ir_2_O_7_ and (Sr_0.8_La_0.2_)_3_Ir_2_O_7_, corresponding to local refined La concentrations of approximately *x* ≈ 0.1 and *x* ≈ 0.2, respectively. These values exceed the La concentrations previously reported for ambient-pressure-grown crystals,^11,13–18^ demonstrating that high-pressure synthesis effectively expands the accessible electron-doping range in the bilayer iridate system. In addition to the large increase in La content, the average crystal structure remains remarkably robust. The lattice parameters of the two selected crystals reported in Table 1 change only slightly from *a* = 3.90868(11) Å and *c* = 20.8717(9) Å for (Sr_0.9_La_0.1_)_3_Ir_2_O_7_ to *a* = 3.91562(13) Å and *c* = 20.8321(9) Å for (Sr_0.8_La_0.2_)_3_Ir_2_O_7_. Consequently, the corresponding room-temperature unit-cell volume increases only slightly from 318.873(19) to 319.40(2) Å^3^ (approximately 0.2%). The slight increase of the in-plane lattice parameter accompanied by a small contraction of the *c*-axis suggests anisotropic structural accommodation of the La dopants while preserving the overall bilayer framework. The oxygen occupancies were not refined freely but were fixed to the values required by the O_7_ structural model, including half occupancy of the split O3 site (16*n*). The identification of the 327 framework by PXRD and the semiquantitative oxygen-to-iridium ratios obtained from representative SEM–EDS spectra are consistent with this model and show no evidence of a large oxygen deficiency, although a small degree of oxygen nonstoichiometry cannot be excluded. Under the O_7_ charge-counting model, the refined compositions correspond to formal average Ir oxidation states of approximately +3.86 and +3.70. Accordingly, La^3+^-for-Sr^2+^ substitution is discussed as formal electron doping rather than as a direct measurement of carrier concentration or Ir valence. Representative SEM-EDS analyses give local A-site La fractions of approximately 0.06–0.07, 0.12, 0.12–0.20, and 0.22–0.41 for the nominal *x* = 0.05, 0.10, 0.15, and 0.20 products, respectively (Table S6 and Figure S1). These semi-quantitative results confirm La incorporation and an overall increase in local La content across the series. Accordingly, the SCXRD compositions are reported as local selected-crystal compositions, whereas the magnetic and transport properties below are discussed in terms of the corresponding nominal synthesis products. Moreover, La atoms are found to occupy both crystallographically distinct Sr sites, including the rock-salt-layer Sr1 site (2*a*) and the perovskite-layer Sr2 site (4*e*). In both compositions, La preferentially occupies the Sr1 site located within the rock-salt layer, indicating site-selective incorporation of the dopant. This preferential occupation may play an important role in stabilizing the heavily doped bilayer structure under high-pressure conditions. The stability of the Ir–O network, together with the nearly constant unit-cell volume, suggests that the primary effect of La substitution is electronic rather than crystallographic. Therefore, the changes in magnetic and transport properties discussed below are unlikely to originate from a major structural transformation.

**Figure 2:**
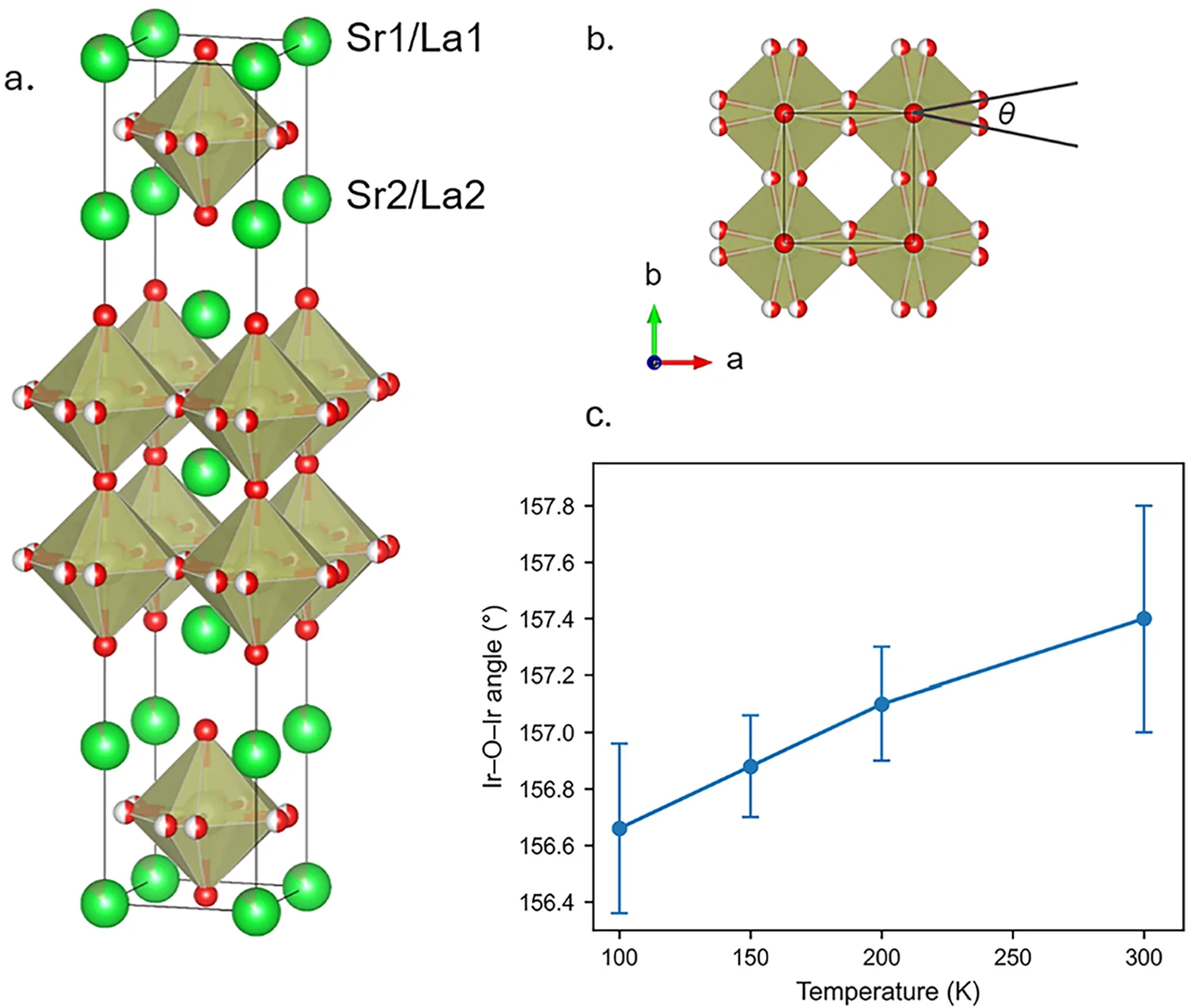
Crystal structure of the high-pressure synthesized bilayer iridate (Sr_1‑*x*_La_*x*_)_3_Ir_2_O_7_ with tetragonal *I*4/*mmm* symmetry. (a) Crystal structure viewed along the *a*-axis, showing the layered Ruddlesden-Popper framework consisting of corner-sharing IrO_6_ octahedral bilayers separated by Sr/La-containing rock-salt layers. The two crystallographically distinct alkaline-earth sites, Sr1 and Sr2, are indicated. (b) Projection of the IrO_6_ octahedral network along the *c*-axis. The angle θ denotes the in-plane Ir–O–Ir bond angle, which governs the octahedral rotation and strongly influences the electronic bandwidth and magnetic exchange interactions in the bilayer iridate. (c) Temperature dependence of the in-plane Ir–O–Ir bond angle for the nominal *x* = 0.05 crystal used in the variable-temperature refinement.

**Table 1:** Single-Crystal X-ray Diffraction Refinement Data for La-Doped (Sr_1–*x*_La_*x*_)_3_Ir_2_O_7_ (*x* = 0.05 and 0.15) at 293 K^a^

Loading Composition	*x* = 0.05	*x* = 0.15
Refined composition	Sr_2.72_La_0.28_Ir_2_O_7_	Sr_2.41_La_0.59_Ir_2_O_7_
Space Group	*I*4/*mmm*	*I*4/*mmm*
*a*(Å)	3.90868(11)	3.91562(13)
*c*(Å)	20.8717(9)	20.8321(9)
*V* (Å^3^)	318.873(19)	319.40(2)
μ (mm^–1^)	66.455	66.083
F(000)	659	670
2q range (°)	7.81 to 73.168	7.826 to 81.228
Reflections collected/Restraints/Parameters	2843/0/23	4496/0/23
Indep. Reflections	280 [*R*_int_ = 0.0265]	347 [*R*_int_ = 0.0327]
Final R indices	*R*_1_(I≥2σ(I)) = 0.0185	*R*_1_(I≥2σ(I)) = 0.0218
	w*R*_2_(I≥2σ(I)) = 0.0420	w*R*_2_(I≥2σ(I)) = 0.0464
	*R*_1_(all) = 0.0223	*R*_1_(all) = 0.0277
	w*R*_2_(all) = 0.0436	w*R*_2_(all) = 0.0477
Goodness-of-fit	1.066	1.090
Δρ_max_(e-/Å^3^)	2.660; −1.40	3.39; −2.36
Crystal size/mm^3^	0.06 × 0.04 × 0.04	0.06 × 0.05 × 0.04
Absorpt. correction	Analytical	Analytical
Transmission*T*_min_/*T*_max_	0.085/0.186	0.061/0.215

a Values in parentheses represent estimated standard deviations from the refinements.

**Table 2:** Atomic Coordinates and Equivalent Isotropic Displacement Parameters (*U*_eq_) for La-Doped (Sr_1–*x*_La_*x*_)_3_Ir_2_O_7_ (*x* = 0.05 and 0.15) Refined from Single-Crystal X-ray Diffraction Collected at 293 K

(Sr_1‑*x*_La_*x*_)_3_Ir_2_O_7_	Atom	Wyck.	*x*	*y*	*z*	Occ.	*U*_eq_ (Å^2^)
Sr_2.72_La_0.28_Ir_2_O_7_	Sr1	2*a*	0	0	0	0.865(13)	0.0141(4)
	La1	2*a*	0	0	0	0.135(13)	0.0141(4)
	Sr2	4*e*	0	0	0.18673(5)	0.928(8)	0.0120(3)
	La2	4*e*	0	0	0.18673(5)	0.072(8)	0.0120(3)
	Ir	4*e*	0	0	0.40251(2)	1	0.00735(12)
	O1	2*b*	0	0	1/2	1	0.023(2)
	O2	4*e*	0	0	0.3053(3)	1	0.0133(13)
	O3	16*n*	0	0.4004(3)	0.0959(16)	1/2	0.0144(11)
Sr_2.41_La_0.59_Ir_2_O_7_	Sr1	2*a*	0	0	0	0.725(12)	0.0148(3)
	La1	2*a*	0	0	0	0.275(12)	0.0148(3)
	Sr2	4*e*	0	0	0.18644(5)	0.843(7)	0.0140(2)
	La2	4*e*	0	0	0.18644(5)	0.157(7)	0.0140(2)
	Ir	4*e*	0	0	0.40246(2)	1	0.00676(8)
	O1	2*b*	0	0	1/2	1	0.034(3)
	O2	4*e*	0	0	0.3046(3)	1	0.0152(13)
	O3	16*n*	0	0.4035(3)	0.0955(16)	1/2	0.0172(13)

### Weak Ferromagnetic-like Transition in the Nominal *x* = 0.05 Product

To investigate the magnetic properties of heavily electron-doped Sr_3_Ir_2_O_7_, temperature-dependent magnetic susceptibility measurements were performed on the nominal *x* = 0.05 product under applied fields of 100 and 1000 Oe, as shown in Figure 3a. A clear magnetic anomaly is observed between 180 and 190 K, indicating the emergence of a ferromagnetic-like ordered state absent in the parent compound.^7–9^ To determine the transition temperature more precisely, the derivative d*M*/d*T* was calculated and is presented in Figure 3b. The minimum in d*M*/d*T* identifies the transition temperature as approximately 186 K. Interestingly, the magnetic transition is most clearly resolved under the lower applied magnetic field of 100 Oe, where the anomaly is observed in both the susceptibility and derivative curves. Upon increasing the applied field to 1000 Oe, the anomaly becomes significantly broader and weaker, indicating that the transition is highly field-sensitive. Such behavior differs from that in a conventional robust ferromagnet, where the transition typically remains well-defined under moderate applied fields. Instead, the suppression and broadening of the anomaly suggest the presence of a weak ferromagnetic or canted magnetic state, which is potentially associated with domain effects, spin canting, or field-induced spin reorientation. The observed field dependence demonstrates that the magnetic order is relatively fragile and likely arises from competing magnetic interactions introduced by heavy electron doping or other couplings.

**Figure 3:**
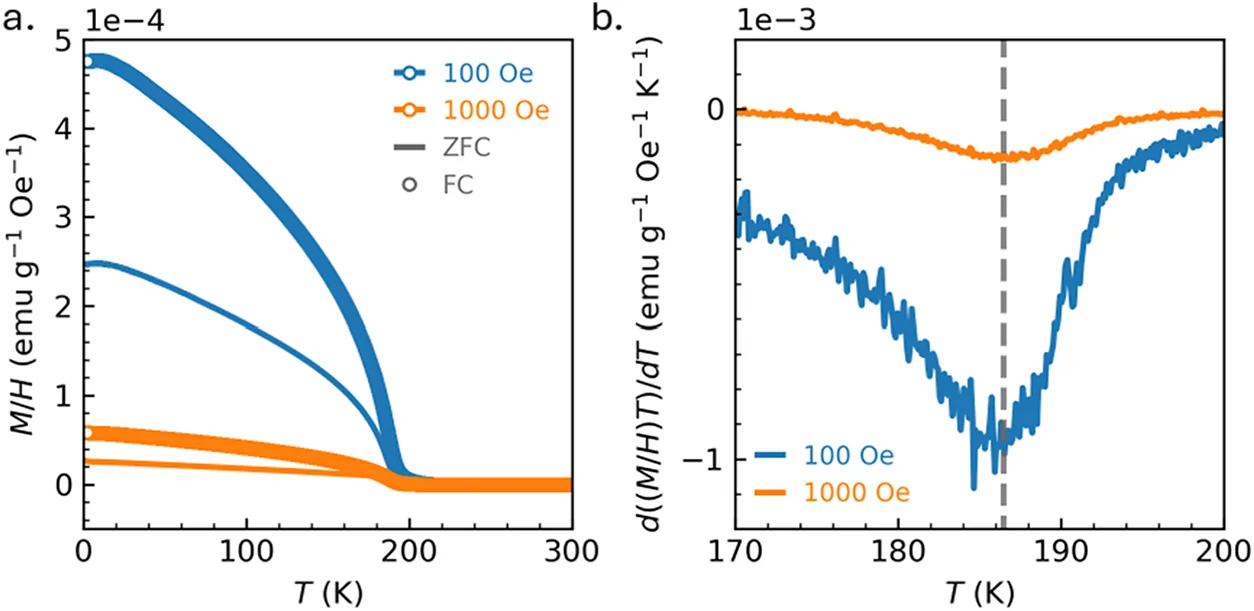
(a) Temperature-dependent magnetic susceptibility of the nominal *x* = 0.05 product measured under applied fields of 100 and 1000 Oe in zero-field-cooled (ZFC) and field-cooled (FC) modes. (b) d*M*/d*T* is used to determine the transition temperature.

To further investigate the nature of the magnetic transition, field-dependent magnetization measurements were performed at selected temperatures spanning the ordered and paramagnetic regions, as shown in Figure 4. At temperatures well below the transition, the magnetic response is relatively weak. The M­(H) curve in Figure 4a at 2 K is nearly linear, exhibiting little evidence of ferromagnetic order. A small hysteresis loop emerges at 50 K, indicating the presence of a weak ferromagnetic component, although the remanent magnetization and coercive field remain small. As the temperature approaches the magnetic transition, the ferromagnetic-like component becomes significantly enhanced. At 100 K, a pronounced hysteresis loop develops with a remanent magnetization of approximately 0.002 μ_B_ per Ir and a coercive field of roughly 1.8 T in Figure 4b. A similarly large hysteretic response is observed at 150 K, indicating that the magnetic state is strongest in the intermediate temperature range below the transition (Figure 4c). At 170 K in Figure 4d and 180 K in Figure 4e, the hysteresis remains clearly visible but is substantially reduced, consistent with the approach to the transition temperature near 186 K. Above the transition temperature shown in Figure 4f, the ferromagnetic-like response is rapidly suppressed. The hysteresis loop observed at 180 K nearly disappears at 190 K, and the magnetization becomes predominantly linear with field. By 300 K, no measurable hysteresis is detected, confirming the absence of long-range magnetic order in the high-temperature state. The abrupt collapse of the hysteretic response across the transition temperature is consistent with the magnetic anomaly observed in the temperature-dependent susceptibility measurements and supports the existence of a field-sensitive weak ferromagnetic or canted magnetic state in the nominal *x* = 0.05 product.

**Figure 4:**
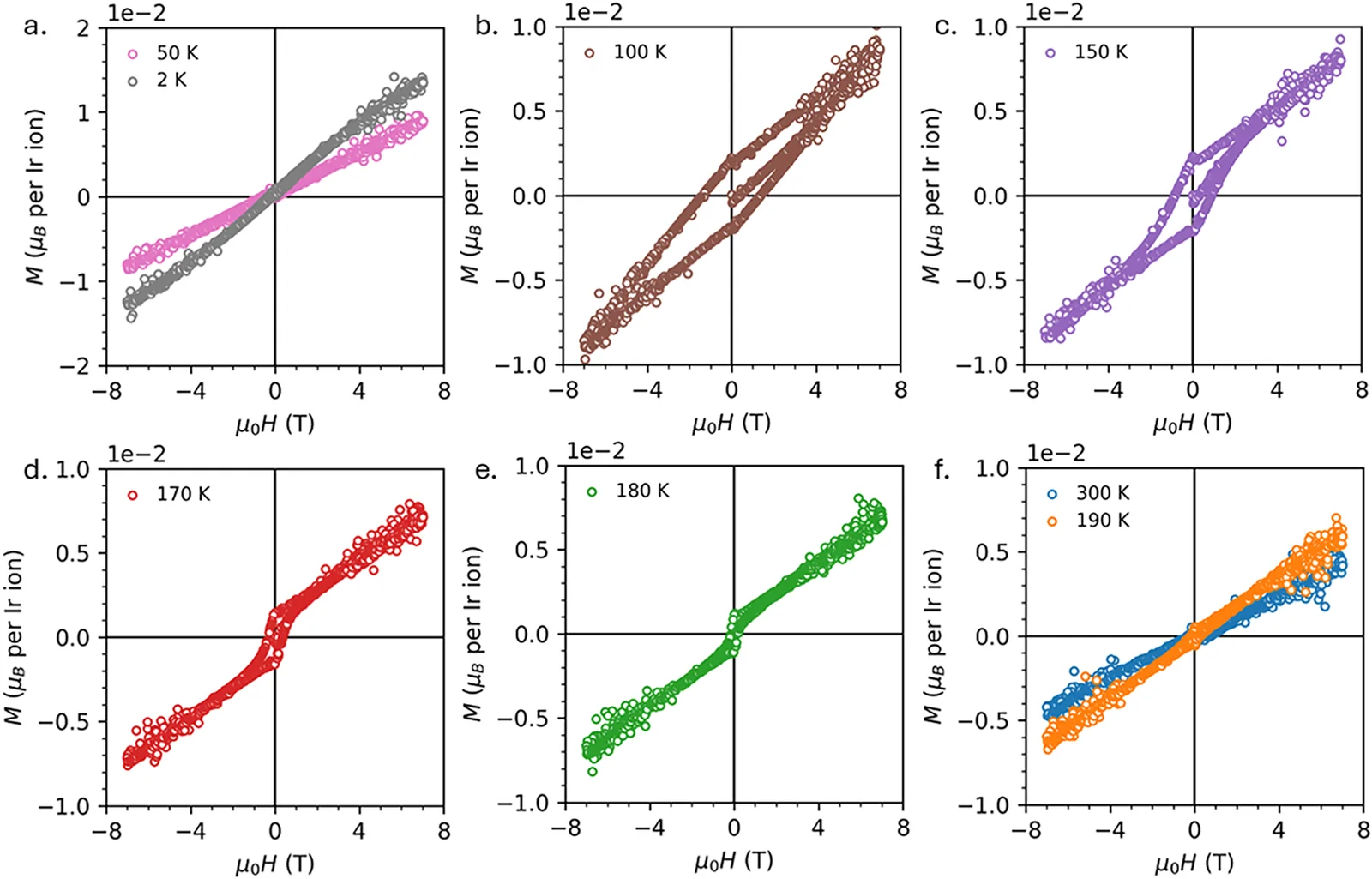
Field-dependent magnetization, M­(H), of the nominal *x* = 0.05 product measured at selected temperatures. (a) M­(H) curves at 2 and 50 K. (b–e) M­(H) curves at 100, 150, 170, and 180 K showing clear hysteresis below the magnetic transition. (f) M­(H) curves at 190 and 300 K, where the ferromagnetic-like response is strongly suppressed. The largest hysteresis is observed between 100 and 150 K, while the response becomes nearly linear above the transition temperature of approximately 186 K.

### Localized Charge Transport in the Nominal *x* = 0.05 Product

The electrical resistance of the nominal *x* = 0.05 product was measured under applied magnetic fields of 0 and 1 T, as shown in Figure 5a. The resistance increases rapidly upon cooling, indicating strongly insulating behavior over the entire measured temperature range. The 0 and 1 T curves nearly overlap, demonstrating that the transport is essentially field-independent. This absence of measurable magnetoresistance suggests that the charge carriers remain strongly localized and that the ferromagnetic-like magnetic transition does not significantly modify the dominant conduction pathway. To examine possible transport anomalies near the magnetic transition, d*R*/d*T* was calculated around 170-200 K, as shown in Figure 5b. Only a subtle change in slope is observed near 186 K, consistent with weak coupling between the magnetic transition and charge transport. Arrhenius analysis of the resistance reveals two thermally activated regimes separated by the magnetic transition (Figure 5c). Above 186 K, the activation energy is approximately 68.40(1) meV, whereas below the transition it increases to 82.42(2) meV. The larger activation energy below the magnetic transition indicates that the ordered magnetic state further enhances carrier localization. These results show that the nominal *x* = 0.05 product is a heavily electron-doped magnetic insulator in which spin ordering is coupled to carrier localization but does not produce field-tunable transport.

**Figure 5:**
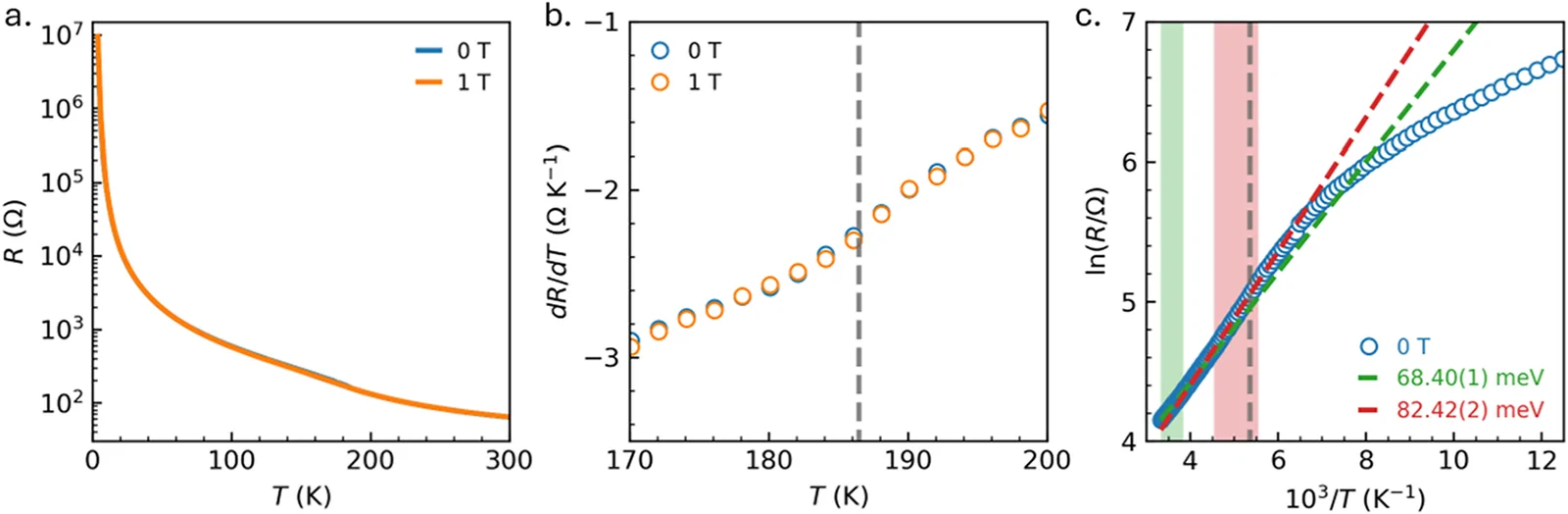
Electrical transport of the nominal *x* = 0.05 product. (a) Temperature-dependent resistance measured under 0 and 1 T, showing strongly insulating behavior with negligible field dependence. (b) d*R*/d*T* near the magnetic transition region, showing only a weak slope change around 186 K. (c) Arrhenius analysis, ln­(*R*) versus 10^3^/T, showing two thermally activated regimes with activation energies of 68.40(1) meV above the transition and 82.42(2) meV below the transition.

### Weak Spin–Lattice Coupling Observed in the (Sr_0.9_La_0.1_)_3_Ir_2_O_7_ Crystal across the Magnetic Transition

To investigate the relationship between the crystal structure and the ferromagnetic-like transition observed near 186 K, temperature-dependent single-crystal X-ray diffraction measurements were performed on (Sr_0.9_La_0.1_)_3_Ir_2_O_7_ between 100 and 300 K on a second crystal independently selected from the same nominal *x* = 0.05 (Sr_1–*x*_La_*x*_)_3_Ir_2_O_7_ product. This crystal was distinct from the crystal used for the room-temperature refinement reported in Tables 1 and 2. Both crystals adopt the same tetragonal *I*4/*mmm* bilayer structure and yield comparable local La substitution levels around *x* ≈ 0.1, supporting the crystal-to-crystal reproducibility of the structural assignment. At all measured temperatures, the structure remains tetragonal with space group *I*4/*mmm*, indicating that the magnetic transition is not accompanied by a crystallographic symmetry-breaking structural transition. The refined lattice parameters and unit-cell volumes are summarized in Tables S4 and S5. As expected, the unit-cell volume increases monotonically with increasing temperature due to thermal expansion. However, the thermal expansion is not uniform throughout the measured temperature range. Between 100 and 150 K, the volume increases by only 0.02 Å^3^, whereas substantially larger increases of 0.472 and 0.459 Å^3^ are observed between 150–200 K and 200–300 K, respectively. The enhanced expansion occurring in the 150–200 K interval coincides with the magnetic transition temperature of approximately 186 K, suggesting a subtle magnetoelastic anomaly associated with the onset of magnetic order. Although no discontinuity or symmetry change is observed, the anomalous thermal expansion indicates that the magnetic state is coupled to the lattice. To further probe the origin of this anomaly, the local geometry of the IrO_6_ octahedral framework was examined, as shown in Figure 2b. The in-plane Ir–O–Ir bond angle increases systematically from 156.66° at 100 K to 156.88° at 150 K, 157.10° at 200 K, and 157.40° at 300 K, corresponding to a gradual reduction of the octahedral rotation with increasing temperature. Notably, the larger changes in the bond angle occur across the magnetic transition region. Because the Ir–O–Ir bond angle directly controls both the electronic bandwidth and magnetic exchange interactions in layered iridates, the observed evolution indicates that the magnetic transition is accompanied by a subtle modification of the IrO_6_ network.^26–28^ Below the transition, the smaller Ir–O–Ir angle corresponds to enhanced octahedral rotation and reduced orbital overlap, consistent with the larger activation energy extracted from transport measurements. The combined evolution of the unit-cell volume and Ir–O–Ir bond angle provides direct evidence for weak spin–lattice coupling in the nominal *x* = 0.05 crystal. While the structural response is modest and does not involve symmetry breaking, the coincidence of the lattice anomalies with the magnetic transition demonstrates that the ferromagnetic-like state is coupled to distortions of the IrO_6_ framework. Such coupling may contribute to the enhanced carrier localization observed below the transition and highlights the important role of lattice degrees of freedom in stabilizing the heavily electron-doped magnetic insulating state.

### Suppression of the Magnetic Insulating State in the Nominal *x* = 0.15 Product

To understand the evolution of the magnetic insulating state with increasing electron doping, the magnetic and transport properties of the nominal *x* = 0.15 product were investigated and compared with those of the nominal *x* = 0.05 product. As shown in Figure 6a, the temperature-dependent magnetic susceptibility exhibits predominantly paramagnetic behavior over the entire measured temperature range. In contrast to the nominal *x* = 0.05 product, no ferromagnetic-like transition is observed near 186 K, indicating that the magnetic state stabilized in the lower-doped sample is completely suppressed at higher La concentrations. A weak low-temperature upturn is present below approximately 30 K and is likely associated with the minor impurity phase detected in the powder X-ray diffraction measurements. The field-dependent magnetization measurements further support the absence of long-range magnetic order. As shown in Figure 6b, the M­(H) curves are nearly linear at 300, 150, and 50 K, consistent with paramagnetic behavior. At 20 K, a slight deviation from linearity and a weak hysteretic component are observed, which likely originate from the impurity phase rather than the intrinsic phase. Unlike the nominal *x* = 0.05 product, no substantial remanent magnetization or coercive field is detected. The temperature-dependent resistance measurements reveal a markedly different electronic state compared with the heavily doped magnetic insulator. As shown in Figure 6c, the resistance decreases with decreasing temperature at high temperatures, indicative of metallic or semimetallic transport. Upon further cooling, a broad minimum develops and the resistance increases at low temperatures, suggesting a crossover to a weakly localized state. In contrast to the nominal *x* = 0.05 product, which remains strongly insulating throughout the measured temperature range, the nominal *x* = 0.15 product exhibits substantially enhanced carrier delocalization. These results indicate that increasing La incorporation suppresses the ferromagnetic-like insulating state and drives the system toward a more itinerant electronic regime, although complete metallic behavior is not achieved due to persistent disorder and localization effects.

**Figure 6:**
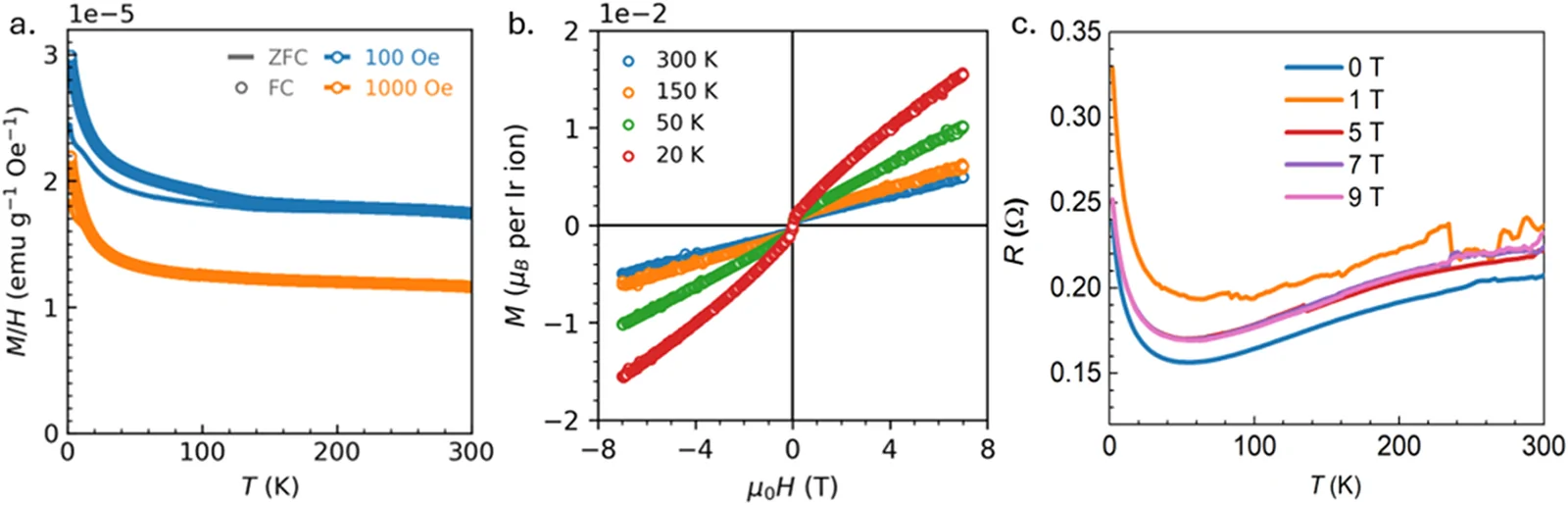
Physical properties of the nominal *x* = 0.15 product. (a) Temperature-dependent magnetic susceptibility measured under applied fields of 100 and 1000 Oe in zero-field-cooled (ZFC) and field-cooled (FC) modes. (b) Field-dependent magnetization measured at selected temperatures, showing predominantly paramagnetic behavior. (c) Temperature-dependent resistance measured at magnetic fields from 0 to 9 T.

## Conclusion

High-pressure high-temperature synthesis extends the experimentally reported La-doping range in the bilayer iridate Sr_3_Ir_2_O_7_. Single-crystal X-ray diffraction reveals that nominal compositions of *x* = 0.05 and 0.15 correspond to approximate local compositions of (Sr_0.9_La_0.1_)_3_Ir_2_O_7_ and (Sr_0.8_La_0.2_)_3_Ir_2_O_7_, respectively, while further La loading drives a structural transformation to cubic perovskite Sr_0.4_La_0.6_IrO_3_. The nominal *x* = 0.05 product exhibits an unexpected ferromagnetic-like transition near 186 K together with strongly insulating transport and weak spin–lattice coupling, establishing a heavily electron-doped magnetic insulating state distinct from both parent Sr_3_Ir_2_O_7_ and previously reported ambient-pressure La-doped samples. In contrast, the nominal *x* = 0.15 product shows paramagnetic behavior and enhanced carrier delocalization, indicating suppression of the magnetic insulating state with increasing electron doping. These results demonstrate that high-pressure synthesis provides a route to previously unreported regions of the electron-doped phase diagram and reveals emergent magnetic and electronic states.

## Supplementary Material



## Notes

Powder XRD Le Bail refinement results for the high-pressure synthesis products; single-crystal X-ray diffraction refinement data and atomic coordinates for the cubic perovskite crystal selected from the nominal *x* = 0.20 product and for a bilayer crystal from the nominal *x* = 0.05 product at 100–300 K; representative SEM–EDS spectra and local cation analyses for the nominal *x* = 0.05, 0.10, 0.15, and 0.20 products (PDF)
