# Supplementary material for: Extending the La Solubility Limit in Sr3Ir2O7 through High-Pressure High-Temperature Synthesis
Source: ACS Mater Au. 2026 Sep 11;6(6):1439–46. doi: 10.1021/acsmaterialsau.6c00140 (PMC13760647; doi:10.1021/acsmaterialsau.6c00140)
Supplement: Supplementary file 1 [file mg6c00140_si_001.pdf]

Supplementary Information

Extending the La Solubility Limit in $Sr_3Ir_2O_7$ through High-Pressure High-Temperature Synthesis

Cheng Peng, Weiwei Xie*

Department of Chemistry, Michigan State University, East Lansing, MI 48864 USA

Corresponding Author: Dr. Weiwei Xie (xieweiwe@msu.edu)

Table of Contents

Table S1. Powder XRD Le Bail refinement results for the high-pressure synthesis products……………S2

Table S2. Single-crystal refinement of cubic perovskite crystals for the nominal x = 0.2 product.....S2

Table S3. Atomic coordinates of cubic perovskite crystals for the nominal x = 0.2 product………...S2

Table S4. Single-crystal refinement of the nominal x = 0.05 crystal from 100 K to 300 K…………...S3

Table S5. Atomic coordinates of the nominal x = 0.05 crystal from 100 K to 300 K………………….S4

Table S6. Representative local SEM-EDS cation ratios…………………………………………...……..S5

Figure S1. Representative SEM-EDS results for La-doped $(Sr_{1-x}La_x)_3Ir_2O_7$ …...........……………………..S6

Table S1. Le Bail refinement results for the high-pressure synthesis products.

Nominal x	Space group	a (Å)	c (Å)	V (Å^3)	R_{wp} (%)
0.05	$I4/mmm$	3.9187	20.9357	321.49	6.577
0.10	$I4/mmm$	3.9106	20.8267	318.50	5.609
0.15	$I4/mmm$	3.9083	20.8235	318.08	8.184

Table S2. Single crystal X-ray diffraction refinement of a cubic perovskite crystal selected from the nominal x = 0.20 product at 293 K.

**Loading Composition**	x = 0.20
**Refined composition**	$Sr_{0.40(7)}La_{0.60(7)}IrO_3$
**Space Group**	Pm-3m
***a* (Å)**	3.9515(5)
**V (Å^3)**	61.70(2)
**μ (mm^{-1})**	92.052
**F(000)**	196
**θ range (º)**	5.160-48.333
**Data/Restraints/Parameters**	2786/0/7
**Indep. Reflections**	89 [R_{int} = 0.0509]
**Final R indices**	R_1(I≥2σ(I)) = 0.0379<br>wR_2(I≥2σ(I)) = 0.0875<br>R_1(all) = 0.0379<br>wR_2(all) = 0.0875
**Goodness-of-fit**	1.179
**$\Delta\rho_{max}$ (e-/Å^3)**	4.041; -4.992

Table S3. Atomic coordinates and equivalent isotropic displacement parameters for nominal $(Sr_{0.8}La_{0.2})_3Ir_2O_7$.

	**Atom**	**Wyck.**	***x***	***y***	***z***	**Occ.**	**U_{eq} (Å^2)**
$Sr_{0.40(7)}La_{0.60}IrO_3$	Sr1	1b	½	½	½	0.40(7)	0.025(1)
	La1	1b	½	½	½	0.60(7)	0.025(1)
	Ir	1a	0	0	0	1	0.0031(4)
	O	3d	0	0	½	1	0.13(2)

Table S4. Single crystal X-ray diffraction refinement for the nominal x = 0.05 $(Sr_{1-x}La_x)_3Ir_2O_7$ sample, at 100 K, 150 K, 200 K and 300 K of a selected crystal size 0.1 × 0.05 × 0.03.

**Temperature**	100 K	150 K	200 K	300 K
**Space Group**	*I*4/*mmm*	*I*4/*mmm*	*I*4/*mmm*	*I*4/*mmm*
***a* (Å)**	3.90117(7)	3.90232(7)	3.90451(7)	3.90864(9)
***c* (Å)**	20.8666(6)	20.8665(6)	20.8740(6)	20.8600(7)
**V (Å^3)**	317.737(12)	317.757(13)	318.229(12)	318.688(15)
**μ (mm^{-1})**	66.624	66.823	66.535	66.496
**F(000)**	660	658	660	659
**2θ range (º)**	7.81 to 82.244	7.812 to 82.234	7.81 to 81.904	7.814 to 82.068
**Reflections collected/Restraints/Parameters**	4686/0/23	4607/0/23	4828/0/23	4575/0/23
**Indep. Reflections**	369 [R_{int} = 0.0489]	367 [R_{int} = 0.0444]	365 [R_{int} = 0.0462]	367 [R_{int} = 0.0619]
**Final R indices**	R_1(I≥2σ(I)) = 0.0150<br>wR_2(I≥2σ(I)) = 0.0366<br>R_1(all) = 0.0177<br>wR_2(all) = 0.0374	R_1(I≥2σ(I)) = 0.0111<br>wR_2(I≥2σ(I)) = 0.0263<br>R_1(all) = 0.0134<br>wR_2(all) = 0.0263	R_1(I≥2σ(I)) = 0.0127<br>wR_2(I≥2σ(I)) = 0.0299<br>R_1(all) = 0.0147<br>wR_2(all) = 0.0303	R_1(I≥2σ(I)) = 0.0187<br>wR_2(I≥2σ(I)) = 0.0479<br>R_1(all) = 0.0215<br>wR_2(all) = 0.0487
**Goodness-of-fit**	1.101	1.122	1.052	1.132
**Δρ$_{max}$ (e-/Å^3)**	2.00; -2.25	1.52; -1.62	1.04; -2.48	3.93; -1.98
**Analytical absorpt. Correction Transmission T$_{min}$/T$_{max}$**	0.064/0.230	0.056/0.233	0.071/0.229	0.028/0.162

Table S5. Atomic coordinates and equivalent isotropic displacement parameters (U_{eq}) for the nominal x=0.05 sample, with an actual refined composition of $(Sr_{0.9}La_{0.1})_3Ir_2O_7$ at 100 K, 150 K, 200 K and 300 K.

$(Sr_{0.9}La_{0.1})_3Ir_2O_7$	**Atom**	**Wyck.**	***x***	***y***	***z***	**Occ.**	**U_{eq} (Å^2)**
100 K	Sr1	2*a*	0	0	0	0.859(9)	0.0086(2)
	La1	2*a*	0	0	0	0.141(9)	0.0086(2)
	Sr2	4*e*	0	0	0.18681(3)	0.919(5)	0.00752(15)
	La2	4*e*	0	0	0.18681(3)	0.081(5)	0.00752(15)
	Ir	4*e*	0	0	0.40252(2)	1	0.00418(7)
	O1	2*b*	0	0	½	1	0.0174(13)
	O2	4*e*	0	0	0.3052(2)	1	0.0085(7)
	O3	16*n*	0	0.3970(11)	0.09595(19)	½	0.0092(6)
150 K	Sr1	2*a*	0	0	0	0.879(6)	0.00901(16)
	La1	2*a*	0	0	0	0.121(6)	0.00901(16)
	Sr2	4*e*	0	0	0.18680(2)	0.927(4)	0.00789(12)
	La2	4*e*	0	0	0.18680(2)	0.073(4)	0.00789(12)
	Ir	4*e*	0	0	0.40252(2)	1	0.00427(5)
	O1	2*b*	0	0	½	1	0.0167(9)
	O2	4*e*	0	0	0.30520(16)	1	0.0080(5)
	O3	16*n*	0	0.3981(8)	0.09584(16)	½	0.0102(5)
200 K	Sr1	2*a*	0	0	0	0.853(7)	0.01041(18)
	La1	2*a*	0	0	0	0.147(7)	0.01041(18)
	Sr2	4*e*	0	0	0.18678(3)	0.915(4)	0.00891(13)
	La2	4*e*	0	0	0.18678(3)	0.085(4)	0.00891(13)
	Ir	4*e*	0	0	0.40252(2)	1	0.00468(6)
	O1	2*b*	0	0	½	1	0.0210(12)
	O2	4*e*	0	0	0.30528(18)	1	0.0096(6)
	O3	16*n*	0	0.3991(9)	0.09594(17)	½	0.0117(6)
300 K	Sr1	2a	0	0	0	0.875(11)	0.0136(3)
	La1	2a	0	0	0	0.125(11)	0.0136(3)
	Sr2	4e	0	0	0.18669(4)	0.917(7)	0.01215(19)
	La2	4e	0	0	0.18669(4)	0.083(7)	0.01215(19)
	Ir	4e	0	0	0.40250(2)	1	0.00672(9)
	O1	2b	0	0	½	1	0.0223(17)
	O2	4e	0	0	0.3054(3)	1	0.0132(9)
	O3	16n	0	0.4003(2)	0.0960(15)	½	0.0146(9)

Table S6. Representative local SEM-EDS cation ratios. The local La fraction is calculated as $x_{\mathrm{EDS}} = \mathrm{La}/(\mathrm{Sr} + \mathrm{La})$. Carbon and oxygen are not included in this A-site ratio. Spectra were selected from Sr–La–Ir–O regions while avoiding visibly distinct impurity phases. Values in parentheses for the nominal x = 0.10 product correspond to spectra collected from secondary-phase regions and are not included in the assessment of the local La content of the dominant 327 phase.

**Nominal x**	**Sample**	**spectra**	$\boldsymbol{x_{\mathrm{EDS}} = \mathrm{La}/(\mathrm{Sr} + \mathrm{La})}$
0.05	TT-1441	13, 15	0.07, 0.06
0.10	BB-1475	1, (5, 10)	0.12, (0.03, 0.16)
0.15	TT-1442	16,19	0.12, 0.20
0.20	TT-1445	20, 21, 26	0.22, 0.31, 0.41

Figure S1. Representative SEM–EDS spectra and backscattered-electron images of the nominal x = 0.05, 0.10, 0.15, and 0.20 synthesis products. The local La fraction was calculated as $x_{\mathrm{EDS}} = \mathrm{La}/(\mathrm{Sr} + \mathrm{La})$. The nominal x = 0.05 product shows relatively uniform local La incorporation, with $x_{\mathrm{EDS}} < 0.10$ in the analyzed regions. The nominal x = 0.10 product contains regions with distinct backscattered-electron contrast and different local cation ratios; Spectra 5 and 10 were collected from secondary-phase regions. One secondary region exhibits an approximately 1:1 $(\mathrm{Sr} + \mathrm{La})$: Ir ratio, consistent with a 113-type perovskite composition, while other regions show different secondary-phase compositions. These chemically distinct regions are consistent with the additional reflections observed in the corresponding PXRD pattern. The nominal x = 0.15 product contains both La-poor and La-rich regions; the specimen selected for magnetic and resistance measurements was taken from a La-rich region. The nominal x = 0.20 product exhibits substantial region-to-region compositional variation.

***x* = 0.05**

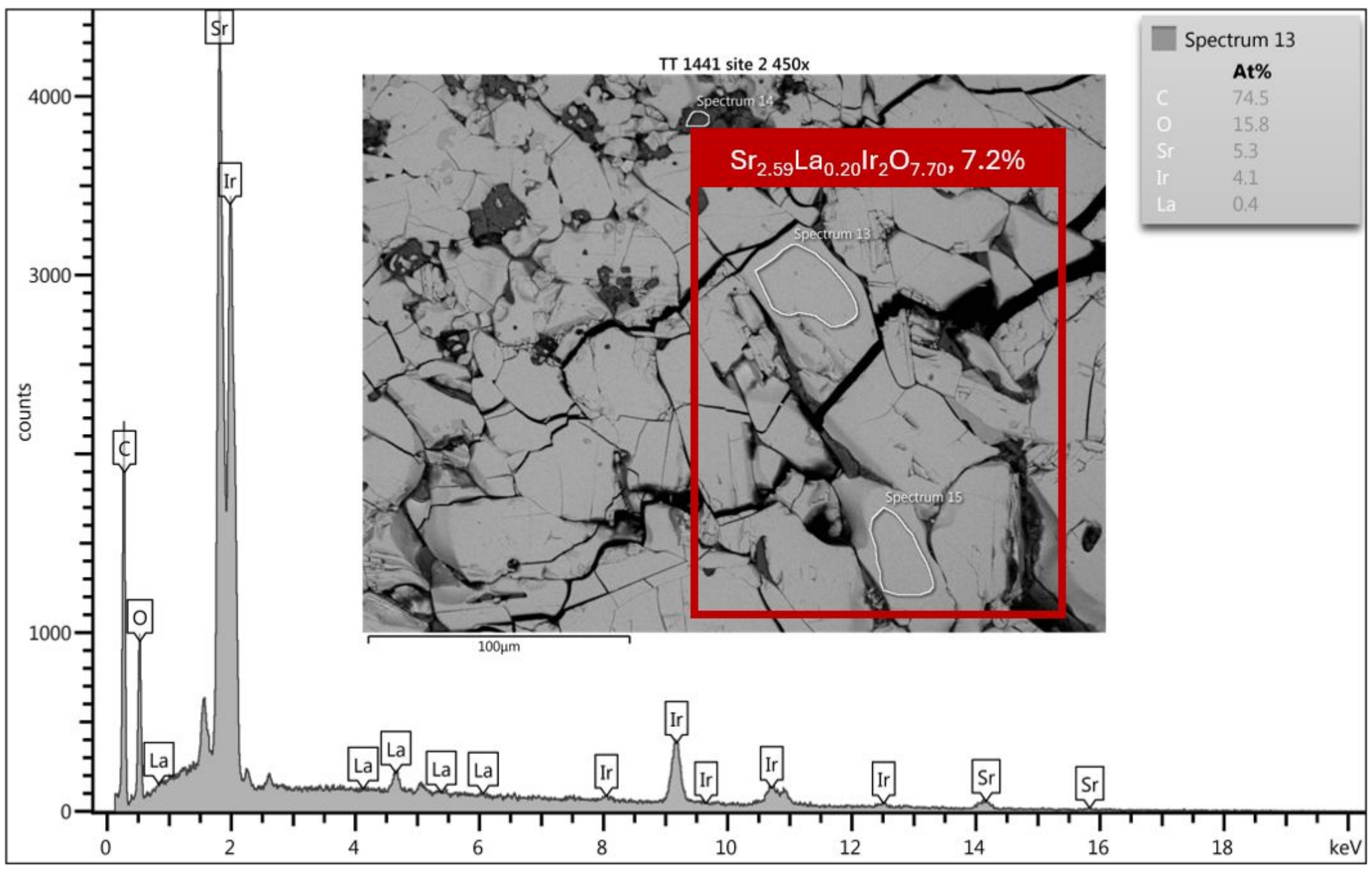

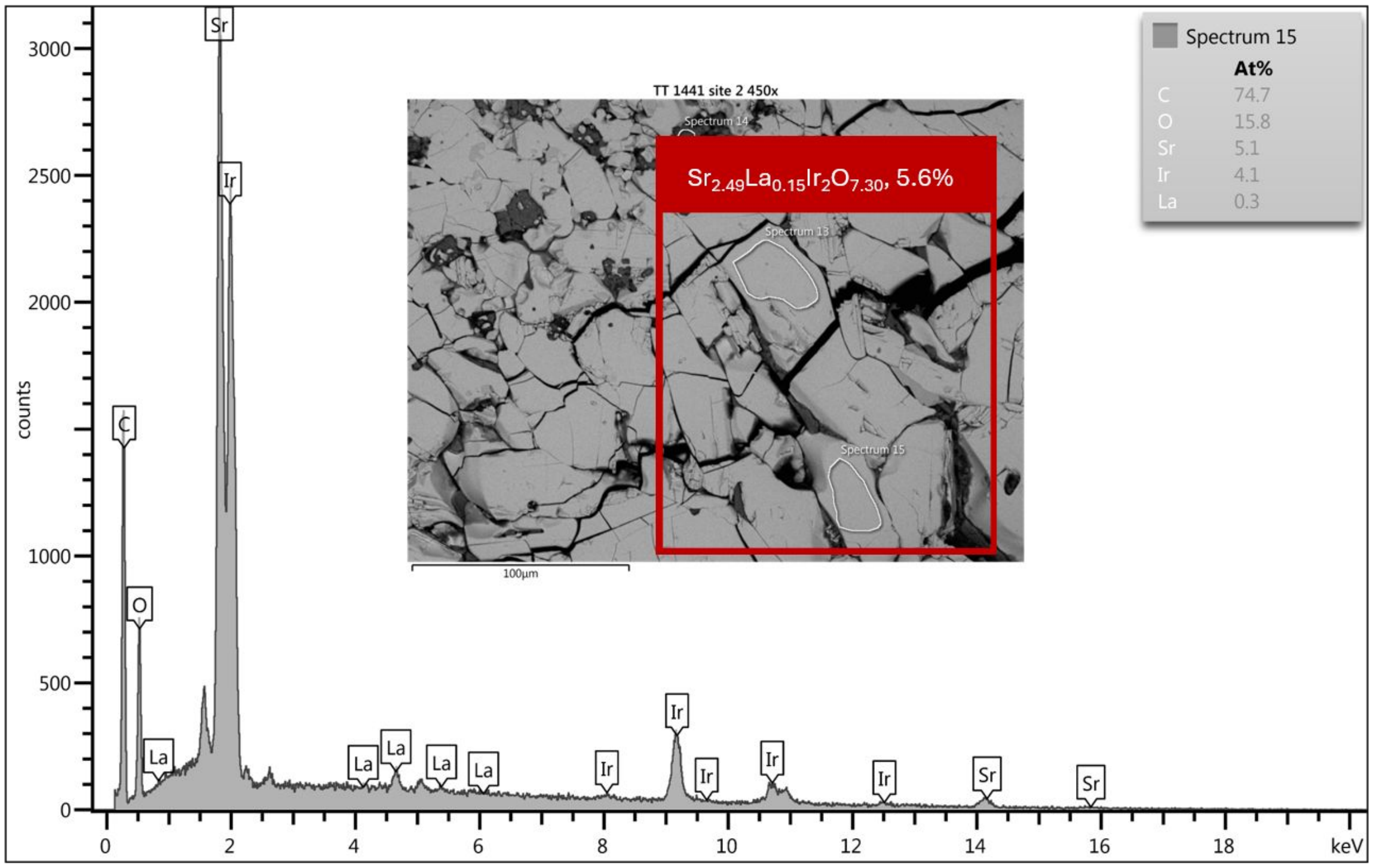
counts
keV
Spectrum 15
At%
C 74.7
O 15.8
Sr 5.1
Ir 4.1
La 0.3
TT 1441 site 2 450x
Sr2.49La0.15Ir2O7.30, 5.6%
100µm

x **= 0.10**

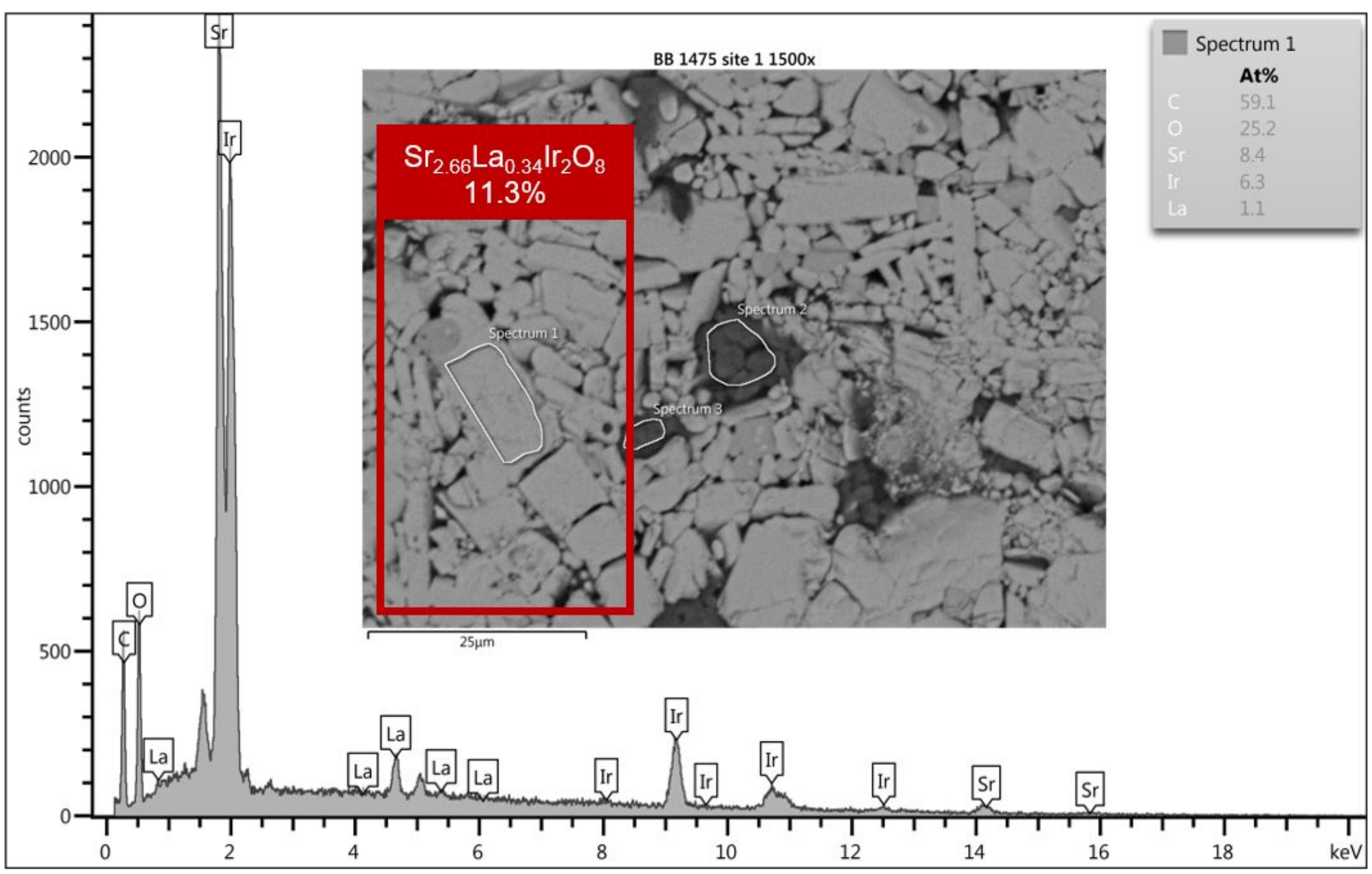
counts
keV
Spectrum 1
At%
C 59.1
O 25.2
Sr 8.4
Ir 6.3
La 1.1
BB 1475 site 1 1500x
Sr2.66La0.34Ir2O8
11.3%
25µm

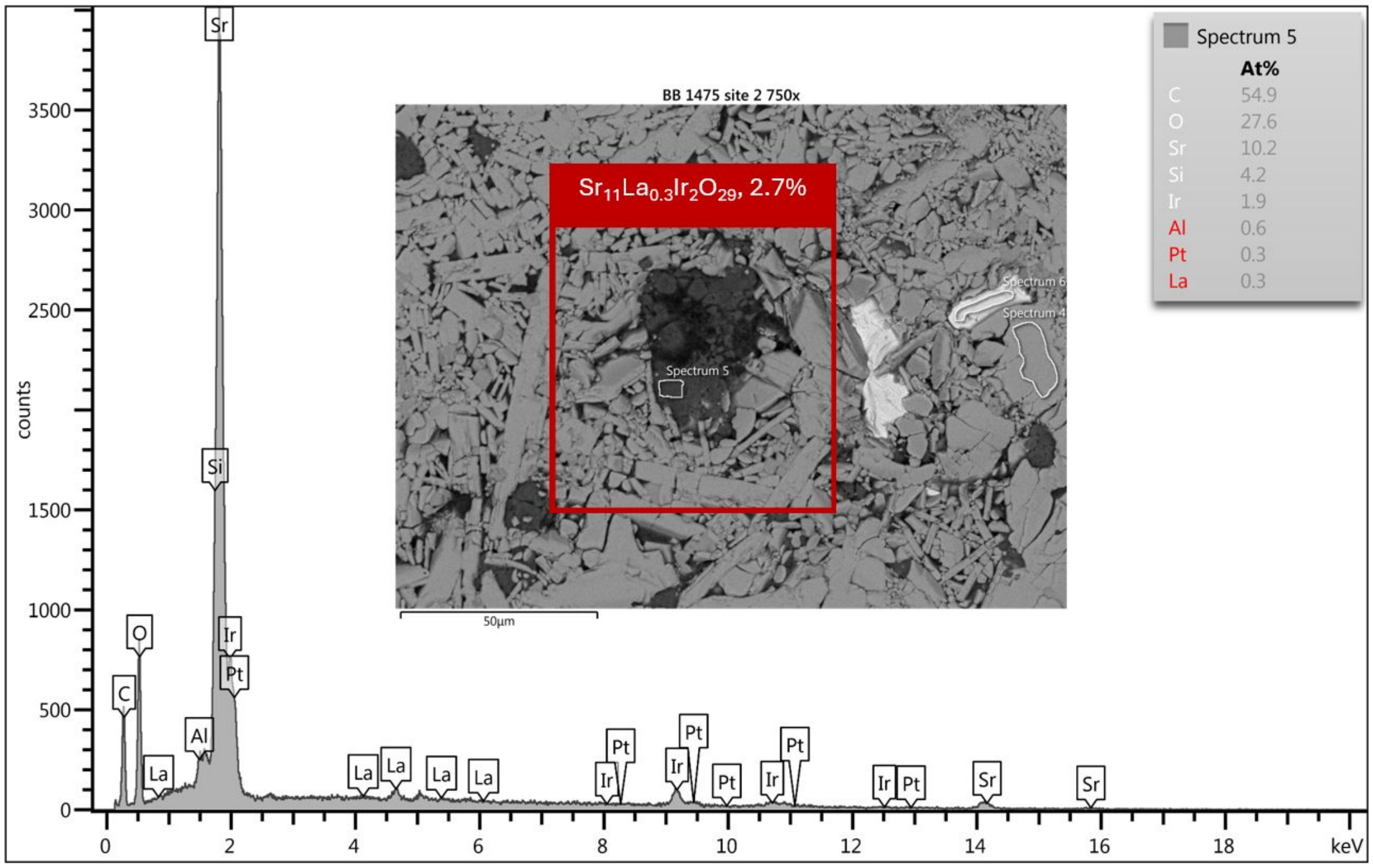

BB 1475 site 2 750x
Sr11La0.3Ir2O29, 2.7%
Spectrum 5
At%
C 54.9
O 27.6
Sr 10.2
Si 4.2
Ir 1.9
Al 0.6
Pt 0.3
La 0.3
50µm
counts
keV

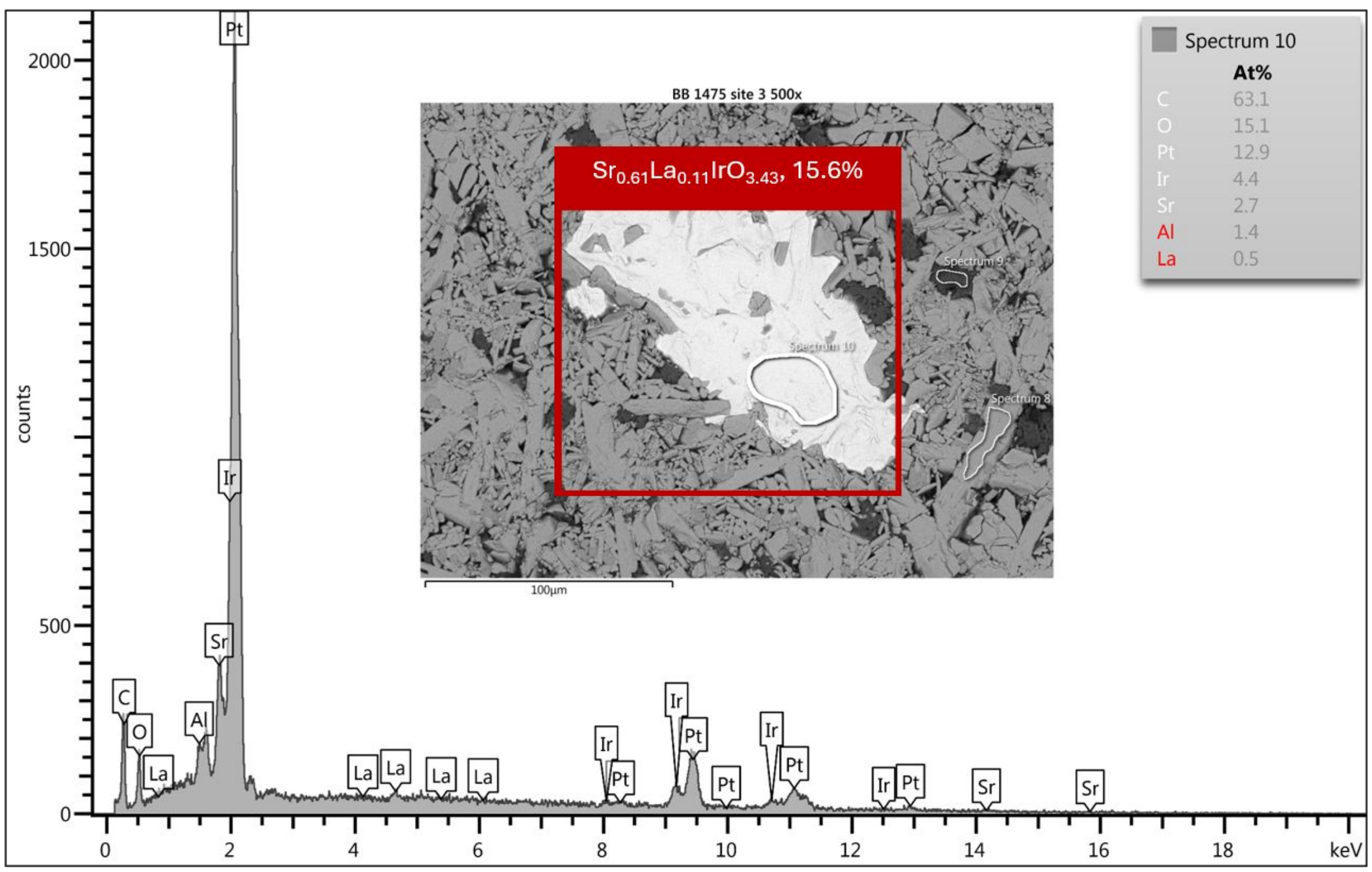

BB 1475 site 3 500x
Sr0.61La0.11IrO3.43, 15.6%
Spectrum 10
At%
C 63.1
O 15.1
Pt 12.9
Ir 4.4
Sr 2.7
Al 1.4
La 0.5
100µm
counts
keV

x = 0.15

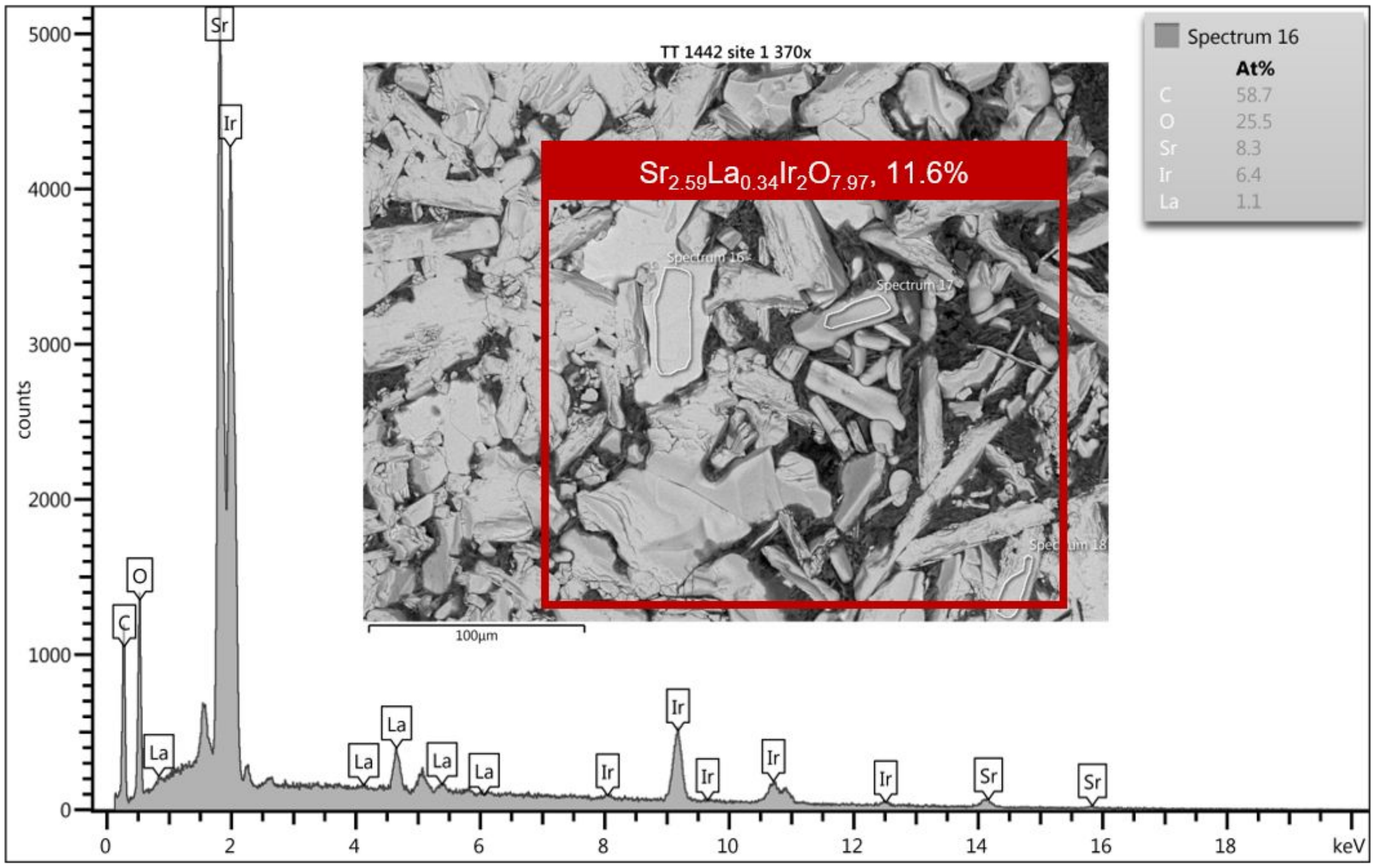

TT 1442 site 1 370x
Sr2.59La0.34Ir2O7.97, 11.6%
Spectrum 16
At%
C 58.7
O 25.5
Sr 8.3
Ir 6.4
La 1.1
100μm
counts
keV

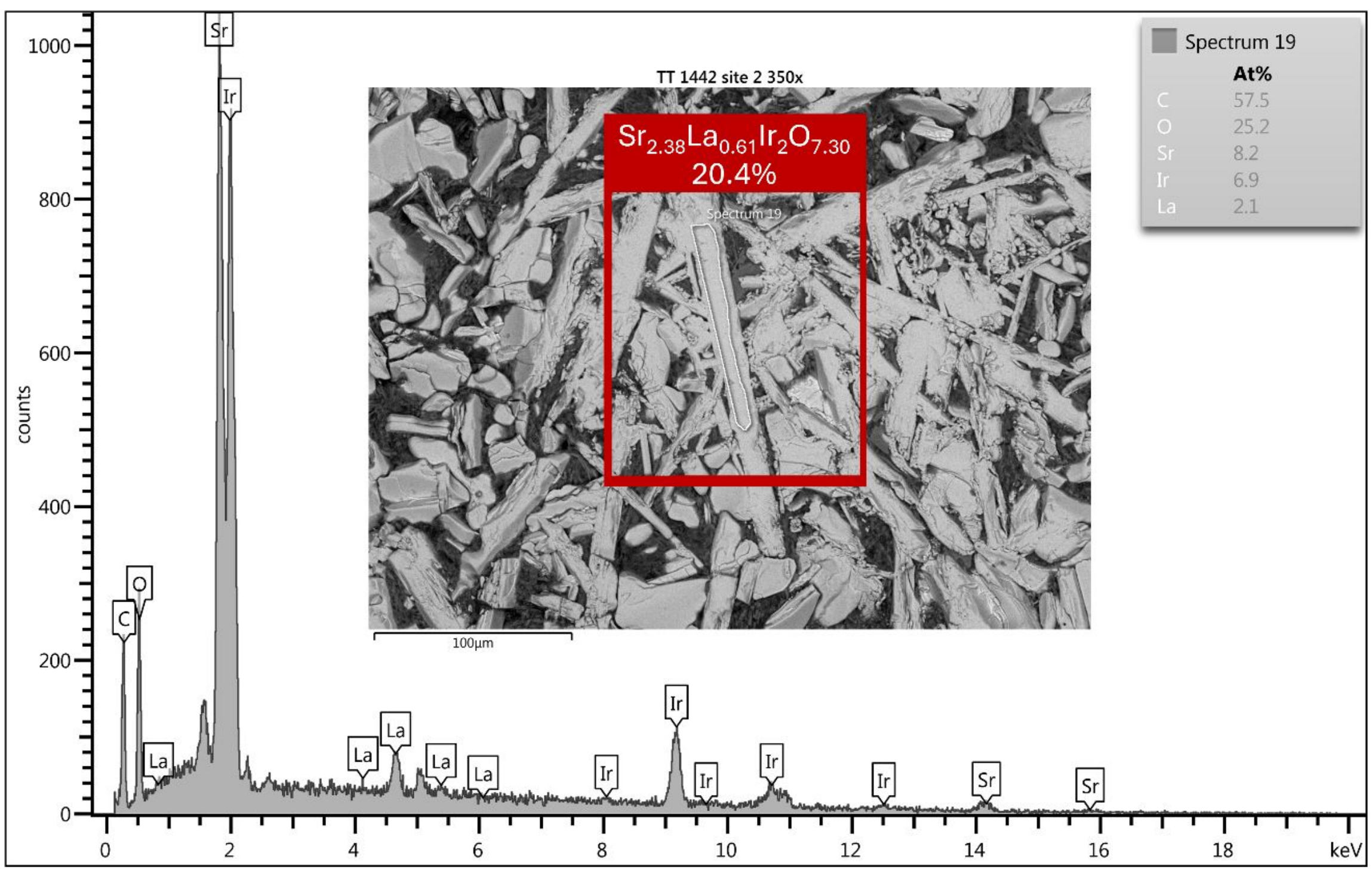

TT 1442 site 2 350x
Sr2.38La0.61Ir2O7.30
20.4%
Spectrum 19
At%
C 57.5
O 25.2
Sr 8.2
Ir 6.9
La 2.1
100μm
counts
keV

x = 0.20

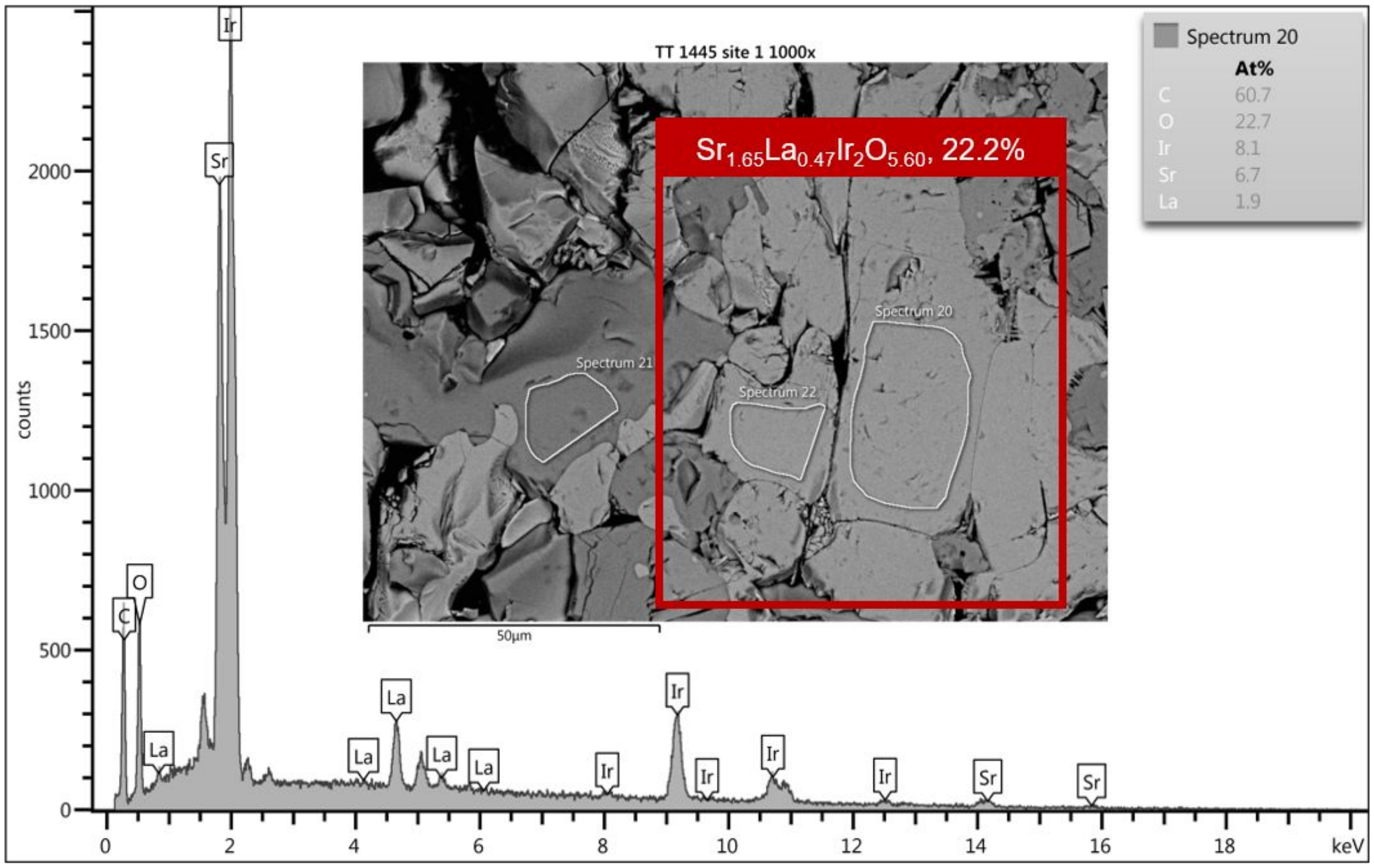
TT 1445 site 1 1000x
$Sr_{1.65}La_{0.47}Ir_2O_{5.60}$, 22.2%
Spectrum 20
Spectrum 21
Spectrum 22
50µm
Spectrum 20
At%
C 60.7
O 22.7
Ir 8.1
Sr 6.7
La 1.9
counts
keV

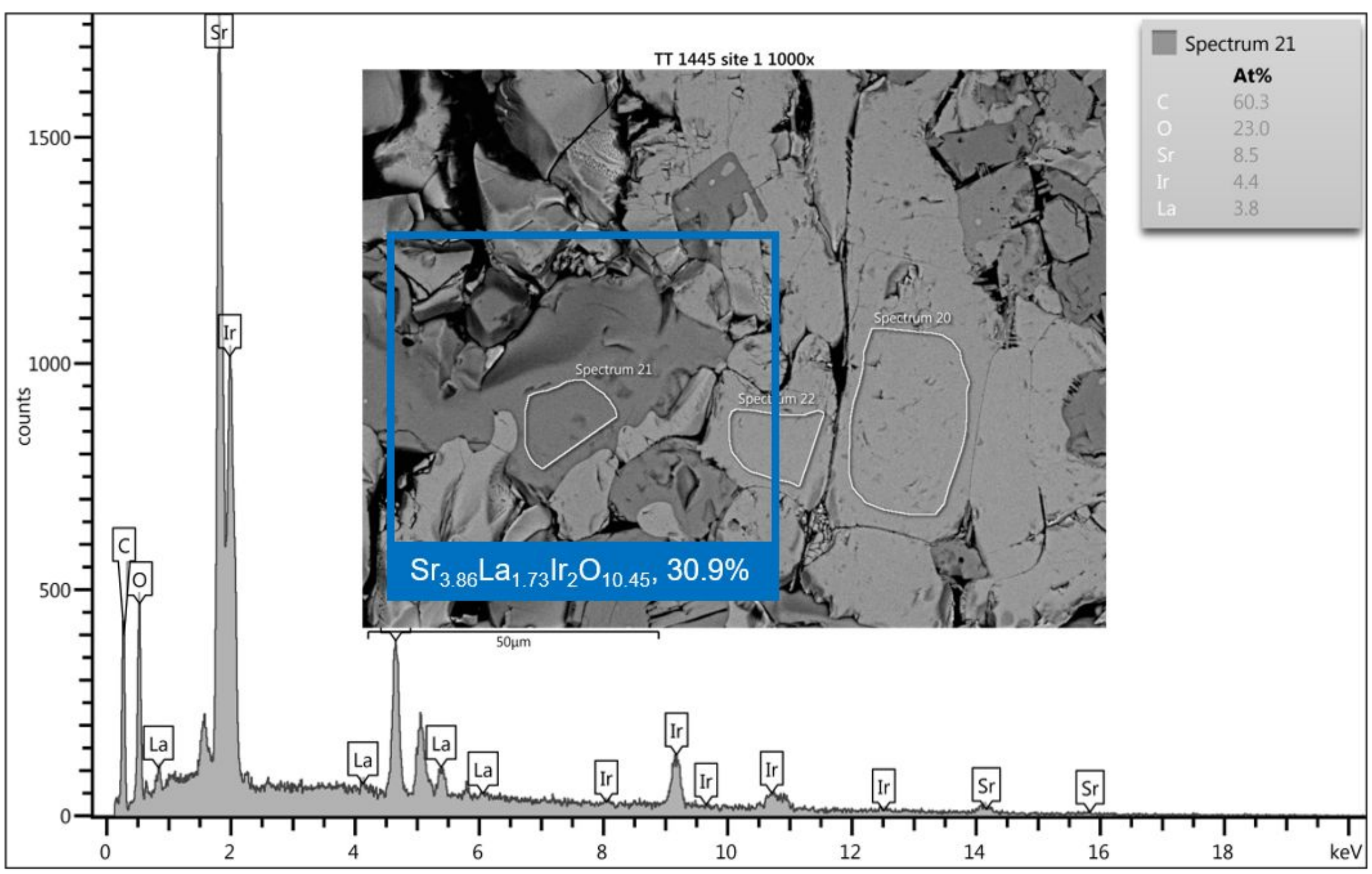
TT 1445 site 1 1000x
$Sr_{3.86}La_{1.73}Ir_2O_{10.45}$, 30.9%
Spectrum 20
Spectrum 21
50µm
Spectrum 21
At%
C 60.3
O 23.0
Sr 8.5
Ir 4.4
La 3.8
counts
keV

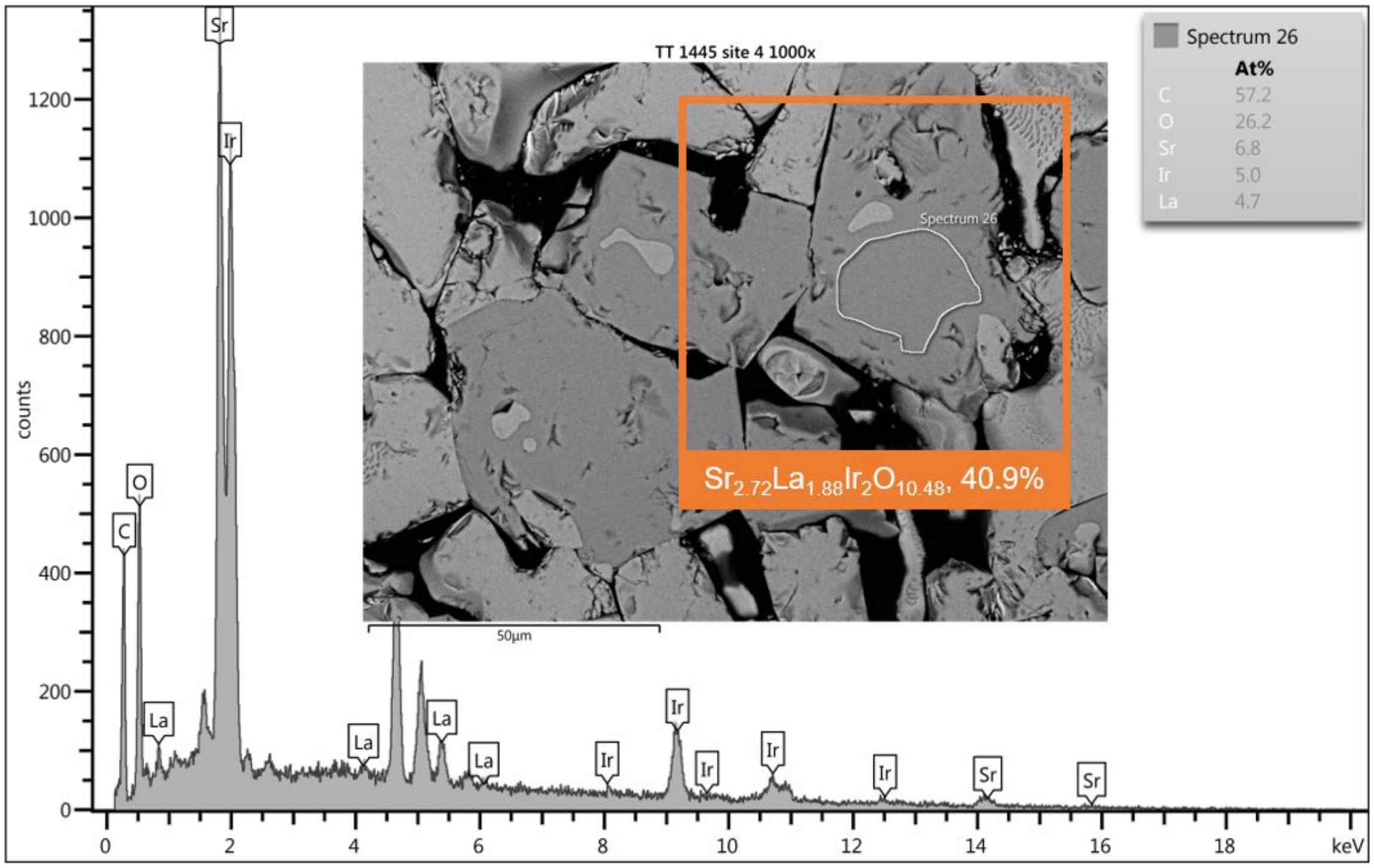

Spectrum 26
At%
C 57.2
O 26.2
Sr 6.8
Ir 5.0
La 4.7
TT 1445 site 4 1000x
Spectrum 26
$Sr_{2.72}La_{1.88}Ir_2O_{10.48}$, 40.9%
50µm
counts
keV
